# An ARID Domain-Containing Protein within Nuclear Bodies Is Required for Sperm Cell Formation in *Arabidopsis thaliana*

**DOI:** 10.1371/journal.pgen.1004421

**Published:** 2014-07-24

**Authors:** Binglian Zheng, Hui He, Yanhua Zheng, Wenye Wu, Sheila McCormick

**Affiliations:** 1 State Key Laboratory of Genetic Engineering, Collaborative Innovation Center for Genetics and Development, Institute of Plant Biology, School of Life Sciences, Fudan University, Shanghai, China; 2 Plant Gene Expression Center, USDA/ARS and Dept. of Plant and Microbial Biology, UC-Berkeley, Albany, California, United States of America; Peking University, China

## Abstract

In plants, each male meiotic product undergoes mitosis, and then one of the resulting cells divides again, yielding a three-celled pollen grain comprised of a vegetative cell and two sperm cells. Several genes have been found to act in this process, and *DUO1* (*DUO POLLEN 1*), a transcription factor, plays a key role in sperm cell formation by activating expression of several germline genes. But how *DUO1* itself is activated and how sperm cell formation is initiated remain unknown. To expand our understanding of sperm cell formation, we characterized an ARID (AT-Rich Interacting Domain)-containing protein, ARID1, that is specifically required for sperm cell formation in *Arabidopsis*. ARID1 localizes within nuclear bodies that are transiently present in the generative cell from which sperm cells arise, coincident with the timing of *DUO1* activation. An *arid1* mutant and antisense *arid1* plants had an increased incidence of pollen with only a single sperm-like cell and exhibited reduced fertility as well as reduced expression of *DUO1*. In vitro and in vivo evidence showed that ARID1 binds to the *DUO1* promoter. Lastly, we found that ARID1 physically associates with histone deacetylase 8 and that histone acetylation, which in wild type is evident only in sperm, expanded to the vegetative cell nucleus in the *arid1* mutant. This study identifies a novel component required for sperm cell formation in plants and uncovers a direct positive regulatory role of ARID1 on *DUO1* through association with histone acetylation.

## Author Summary

For all eukaryotes, gamete formation is an essential aspect of sexual reproduction. Unlike in animals, where meiotic products directly become gametes, the germline in plants is established by two consecutive mitotic divisions after meiosis is completed. The first mitosis is asymmetric, forming a larger vegetative cell and a smaller generative cell. The smaller generative cell then divides to produce two sperm cells. Current knowledge indicates DUO1 (DUO POLLEN 1), a transcription factor, plays a key role in this process by controlling expression of other germline genes. But how *DUO1* is activated in the generative cell is unknown. To better understand the mechanisms that govern sperm cell formation and activate *DUO1* expression, we characterized, *ARID1*, encoding an ARID (AT-Rich Interacting Domain)-containing protein. We show that ARID1 is required for *DUO1* activation and sperm cell formation in *Arabidopsis*. Furthermore, ARID1 physically associates with a histone deacetylase, facilitating the maintenance of histone acetylation between the vegetative nucleus and sperm nuclei. Thus, our study shows that a pollen-specific ARID protein plays an important role during sperm cell formation in a dual manner: as a transcription factor to activate *DUO1* and as a potential component of the histone modification machinery to maintain epigenetic status in pollen.

## Introduction

In contrast to animals, where meiotic products directly become gametes, the germline in plants is established by mitotic divisions after meiosis is completed. The male germline arises by an asymmetric mitotic division of each meiotic product. The resulting vegetative and generative cells of the bicellular pollen grain have distinct fates. The larger vegetative cell is arrested at the G1 phase of the cell cycle, while the smaller generative cell divides mitotically to produce the two male gametes or sperm cells [1]. The vegetative cell forms a pollen tube to convey the sperm cells to the female gametes. Several genes have been implicated in sperm cell formation [2]–[8]. Among these, *DUO POLLEN1* (*DUO1*) encodes a male germ cell–specific R2R3 Myb transcription factor that is necessary for twin sperm cell formation [3].

Although many genes have been implicated in sperm cell formation, the molecular mechanism as to how the generative cell initiates the second mitosis and divides into two sperm cells remains unclear. By monitoring the expression of genes associated with germ cell specification, a previous study [9] demonstrated that DUO1 controls germline expression of a cyclin, *CYCB1*, and dictates correct differentiation of the male germline by promoting expression of *MGH3* (*MALE GAMETE-SPECIFIC HISTONE H3*) [2], *GCS1* (*GENERATIVE CELL SPECIFIC 1*) [5], and *GEX2* (*GAMETE EXPRESSED 2*) [10]. Subsequently, they profiled *DUO1* targets at a genome-wide level [11]. In contrast to the myriad *DUO1* targets, how *DUO1* transcription is regulated is less understood. Only microRNA159 (miR159) was found to inhibit *DUO1* expression [12]. miR159 is greatly reduced but not absent at the bicellular stage [8], but *DUO1* is gradually activated from the early bicellular stage to the middle bicellular stage [9], indicating that *DUO1* activation is not due only to the decrease of miR159 at the bicellular stage and that other factors are required for *DUO1* activation during sperm cell formation. However, such factors were unknown.

In animals, ARID (AT-Rich Interacting Domain) proteins exhibit a range of cellular functions, including participation in epigenetic regulation of gene expression during cell differentiation and development [13]. ARID is an ancient DNA-binding domain that is conserved throughout higher eukaryotes. In animals, ARID proteins are grouped in several subfamilies based on the presence of additional conserved domains [13]. In mice, ARID4A and ARID4B are required for male fertility [14], [15] and are associated with the histone deacetylase complex [16], [17]. JARID2, an important developmental regulator during cell cycle regulation [18], [19], is also a component of the Polycomb-repressive complex 2 (PRC2) either as an activator [20], [21] or a repressor [22], [23] in mammals. Thus, considering the importance of the cell cycle during sperm cell formation in plants and the increasing knowledge about the role of ARID domain-containing proteins both in gene regulation and germline development, we wanted to know whether plant ARID proteins might be involved in the regulation of sperm cell formation. The *Arabidopsis* genome encodes 10 ARID proteins that have been grouped into four subfamilies based on the presence of domains located at the C-terminus [24]. However, no function of these ARID proteins has been reported. In lotus, an ARID is required for early nodule development [24]. Here, we show that ARID1, an *Arabidopsis* pollen-specific ARID protein containing an ELM2 (EGL-27 and MTA1 homology) domain, is required for sperm cell formation, as plants with disrupted *ARID1* function had an increased incidence of pollen with single sperm and exhibited reduced fertility. Furthermore, *DUO1* expression was reduced in *arid1* mutants and Chromatin Immunoprecipitation analysis and in vitro DNA binding assays demonstrated that ARID1 binds to the *DUO1* promoter. Microscopic analysis showed that ARID1 is located in developmentally dynamic nuclear bodies during pollen development, and ARID1 obviously accumulated in the generative cell, which implies that the presence of ARID1 is correlated with the initiation of the second mitosis. We showed that ARID1 physically interacts with histone deacetylase 8 (HDA8) in vitro and in vivo, and an immunofluorescence assay showed that the histone acetylation signal expanded to the vegetative nucleus in *arid1* pollen. Furthermore, an obviously reduced level of histone acetylation was observed at *DUO1*, whereas the level of histone methylation was not altered. These results imply that ARID1 is required for sperm cell formation by positively regulating *DUO1* and for maintaining the levels of histone acetylation in pollen by associating with the histone modification machinery.

## Results

### ARID1 Is Required for Sperm Cell Formation in *Arabidopsis*

**Figure 1:**
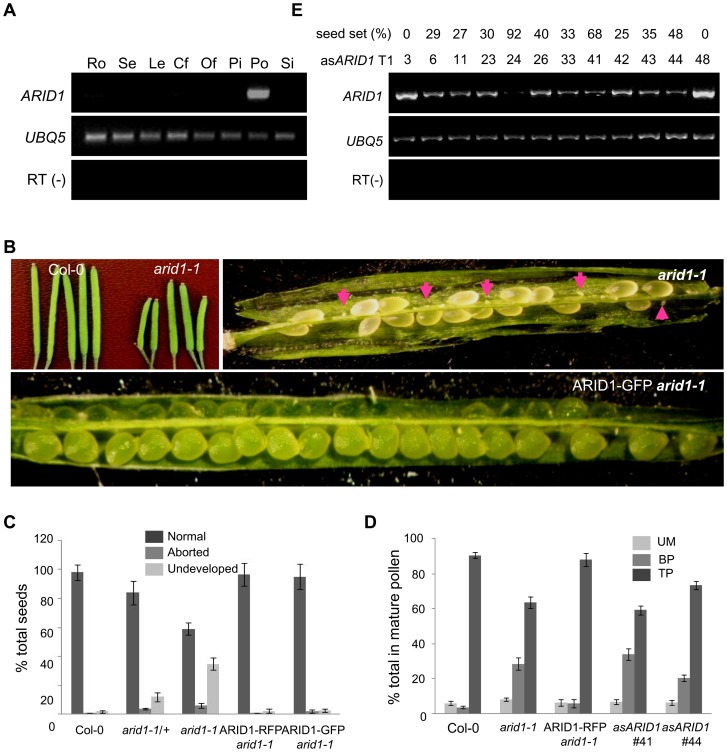
Specific expression of *ARID1* in pollen and disruption of *ARID1* results in defective sperm cell formation. (A) Expression of *ARID1* by RT-PCR. Ro, roots; Se, seedlings; Le, leaves; Cf, closed flower buds; Of, open flowers; Pi, unpollinated pistils; Po, mature pollen; Si, siliques. The RT (−) control PCR was performed with *UBQ5* primers. (B) Representative siliques of WT and *arid1-1* and complementation test. Undeveloped ovules are indicated with arrows. (C) Percentage of normal seeds (dark grey), aborted seeds (lighter grey), and undeveloped ovules (lightest grey) from self-pollinated plants are shown. Error bars represent standard deviation from the mean. (D) Seed set analysis in antisense *ARID1* transgenic plants. Numbers in the bottom row represent individual T1 lines, and the corresponding numbers in the top row indicate the percentage of reduced seed set in each line. The gel shows the expression of *ARID1* as assessed by RT-PCR analysis. (E) Distribution of unicellular microspores (UM, lightest grey), bicellular pollen (BP, lighter grey), and tricellular pollen (TP, dark grey) in mature anthers. At least 600 pollen grains were stained with DAPI and used for statistical analysis; Error bars represent standard deviation from the mean.

Microarray data [25], [26] and our RT-PCR results (Figure 1A) showed that one *ARID*, *At2g46040*, here named *ARID1*, was expressed in a pollen-specific manner. In addition to the ARID domain, ARID1 contains an ELM2 domain at the C-terminus. In animals, ELM2 domains mediate histone modifications by interacting with histone deacetylase [27], [28]. The combination of ARID and ELM2 domains in a single protein is plant-specific. Given the importance of cell cycle regulation by ARID proteins in animals [19], and that a mouse mutant in a gene encoding an ARID protein is male infertile [15], we suspected that disrupting ARID1 function might lead to disorganized cell divisions during pollen development. The *arid1-1* mutant has a T-DNA insertion in the only intron of *ARID1* (Figure S1A). The insertion did not abolish expression, as a truncated transcript upstream of the inserted location was detected (Figure S1A), suggesting that *arid1-1* might be a weak allele. Plants homozygous for *arid1-1* had short siliques and reduced seed set (Figure 1B and 1C). We identified homozygous plants by genotyping a F2 population of *arid1-1* backcrossed with wild type plants. As only 10 of the 96 F2 plants were homozygous, we hypothesized that there might be a transmission problem. Reciprocal crosses with wild type plants showed that transmission through the female was normal, but was perturbed through the male (Table 1). To investigate whether the *arid1-1* phenotype was caused by the T-DNA insertion, we constructed two *ARID1* transgenes by engineering GFP or RFP tags at the C-terminus to a genomic fragment of *ARID1*, driven by its native promoter. ARID1-GFP or ARID1-RFP completely complemented the reduced seed set phenotype (Figure 1B and 1C). Because *arid1-1* appeared to be a weak allele, we explored whether a more complete loss of *arid1* function would have similar or more severe phenotypes. We therefore generated a binary construct expressing antisense *ARID1* under the control of the native *ARID1* promoter. The seed set of 48 independent transgenic lines was examined: 42 plants showed reduced seed set, ranging from 15% to 95% (Figure 1D). Transcript analysis of representative antisense lines confirmed that the phenotype of reduced seed set correlated with reduced transcript levels of *ARID1* (Figure 1D). Because immature seeds from antisense lines with severely reduced seed set finally shriveled, we performed further analyses using *arid1-1*.

**Table 1:** Reduced male transmission in *arid1-1* plants.

Parental genotype ♀×♂	Genotype of F1 plants		Transmission efficiency (TE)
	*arid1-1/+*	wild type	
*arid1-1/+*×WT	182	177	182/177×100% = 102.8%
WT×*arid1-1/+*	181	257	181/257×100% = 70.4%

To dissect which developmental stage was defective in the *arid1-1* mutant and antisense lines, we stained with DAPI to examine pollen development, used in vitro pollen germination assays to observe pollen germination and pollen tube growth, and performed ovule clearing analysis followed by DAB (Decolorized Aniline Blue) staining to evaluate pollen tube guidance. There were no detectable defects in pollen germination and pollen tube growth (Figure S1B) or pollen tube guidance (Figure S1C), but both the *arid1-1* mutant and antisense lines showed increased incidence of single sperm-like cells in mature pollen (Figure 1E and Figure S1D). The stronger phenotypes observed in the antisense *ARID1* transgenic plants suggest that the ARID domain present in truncated proteins in *arid1-1* was partly functional. Because antisense *ARID1* transgenic plants with strongly reduced or undetectable expression of *ARID1* were sterile, we performed all further analyses in the *arid1-1* mutant background. The defect in sperm cell formation was rescued when we introduced the ARID1-RFP construct into the *arid1-1* mutant (Figure 1E). Taken together, our phenotypic analysis indicates that ARID1 is required for sperm cell formation.

### ARID1 Positively Regulates *DUO1* Expression during Sperm Cell Formation

**Figure 2:**
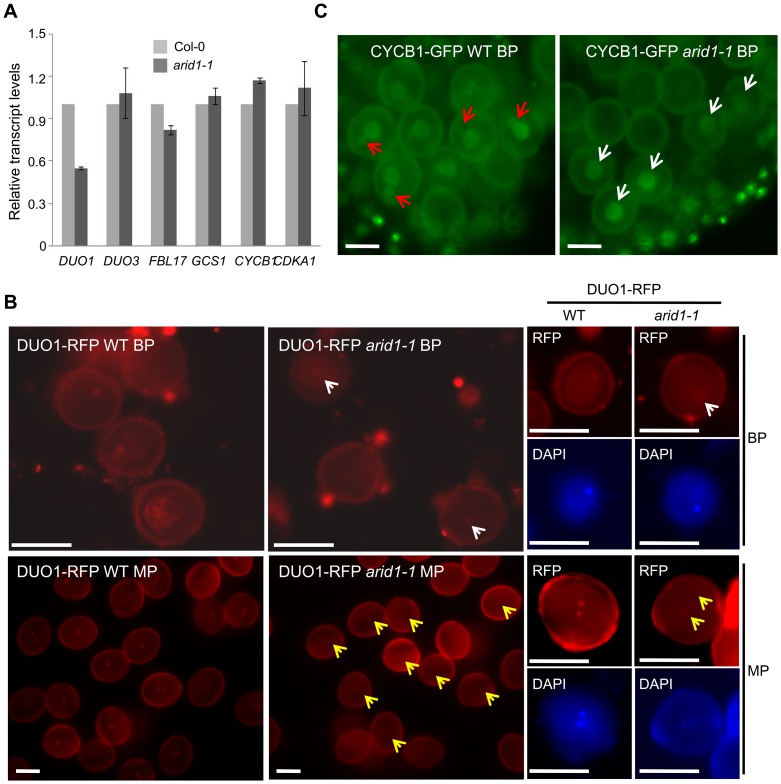
Reduced *DUO1* and CYCB1 expression in *arid1-1*. (A) Expression of sperm-specific genes in mature pollen of wild type and *arid1-1*. Error bars represent the SE from the mean of three biological replicates. (B) Expression of DUO1-RFP in wild type and *arid1-1*. Representative images for each genotype were acquired with the same exposure times. White and red arrows indicate reduced DUO1-RFP signal in bicellular pollen and mature pollen, respectively. Scale bar, 10 µm. (C) Expression of CYCB1-GFP in the bicellular pollen of wild type and *arid1-1*, respectively. The red arrows indicate visible GFP accumulation of CYCB1 in the generative nucleus of wild type pollen, and the white arrows indicate unchanged GFP signal in the vegetative nuclei of the *arid1-1* pollen. Scale bar, 10 µm.

The single sperm-like phenotype of *arid1-1* was similar to the phenotypes of mutants such as *duo1* [3], *cdka1;1* [4], *fbl17* [6], and *duo3* [7]. We therefore used qPCR to examine whether the expression of these genes was disturbed in *arid1-1*. Of these, only *DUO1* mRNA levels were reduced in *arid1-1* (Figure 2A), suggesting that ARID1 positively regulates *DUO1* at the transcriptional level, either directly or indirectly. *DUO1* expression was also reduced in the antisense *ARID1* plants with reduced seed set (Figure S2A). We also crossed a DUO1-RFP reporter into *arid1-1* and saw that the DUO1-RFP signal was slightly reduced, in both bicellular pollen (Figure 2B, upper panels, yellow arrows) and mature pollen (Figure 2B, lower panels, yellow arrows). Since *DUO1* is one of the targets of miR159 [12], we examined *MIR159* expression in the *arid1-1* mutant by qPCR, but found no change in miR159 levels (Figure S2B). As we recently showed that Anaphase Promoting Complex 8 (APC8) is involved in CYCB1 regulation at both the transcriptional level and protein degradation level [8], we crossed APC8-YFP and CYCB1-GFP into *arid1-1*. CYCB1 is mainly present during early pollen developmental stages but not in mature pollen [8], [9]. Overall *CYCB1* expression in *arid1-1* was not altered, as assessed by qPCR (Figure 2B). However, the weak accumulation of CYCB1 in the generative cell of wild type bicellular pollen was undetectable in *arid1-1* (Figure 2C, red arrows), although no apparent change of CYCB1 accumulation in the vegetative cell was seen in *arid1-1* (Figure 2C, white arrows). However, the APC8-YFP level was not affected in *arid1-1* (Figure S2C). Similarly, we detected no effect on the expression of other known genes implicated in sperm cell function (Figure S2D), including *HTR10* [2], *GEX2* [10] and *GEX1* [29]. Taken together, these results suggest that ARID1 might promote *DUO1* expression directly, but independently, of miR159.

### ARID1 Binds to the *DUO1* Promoter

**Figure 3:**
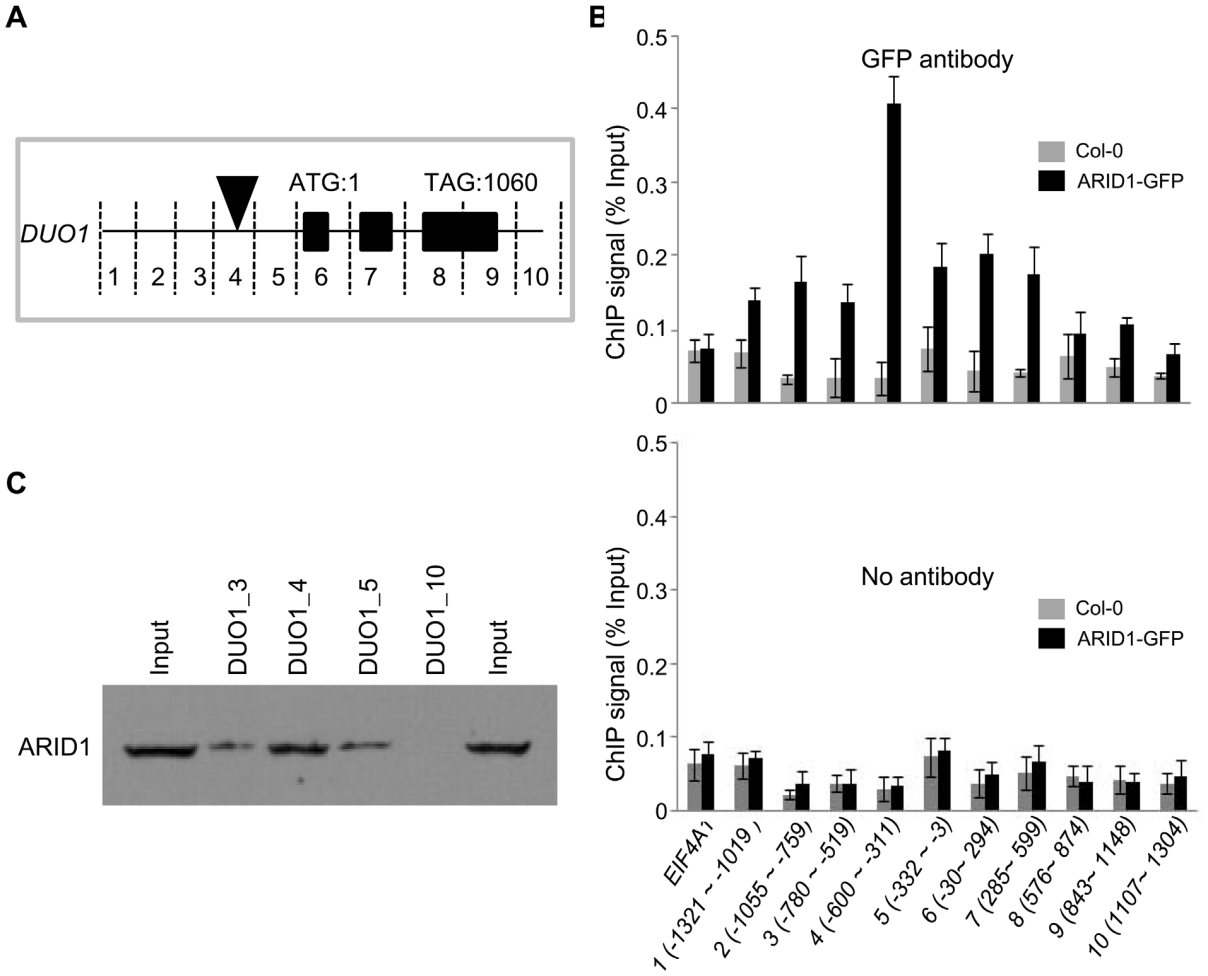
ARID1 binds to *DUO1*. (A) Schematic of subfragments of *DUO1* genomic DNA. The black rectangles represent exons. The black triangle indicates the region where ARID1 was most abundant. The position of the ATG was set to 1, and the fragments upstream or downstream were numbered; for example fragment “1” is −1321 to −1019 bp. (B) ChIP performed with wild type (gray bars) or *ARID1-GFP* (black bars) with GFP antibody (upper panel) and No antibody control (lower panel). *EIF4A1* was used an internal negative control. The results were reproducible in two biological replicates. Error bars show SD calculated from three technical replicates. (C) DNA binding assay. Proteins were resolved by SDS-PAGE and then immunoblotted using anti-ARID1. The input lanes have 1/5 of the amount of ARID1 protein used in the DNA binding assay. The lanes numbered 3, 4, 5 and 10 (corresponding to regions mentioned in (A)) have 1/6 of the DNA-bound protein.

To test how ARID1 affects *DUO1* expression, we performed a ChIP assay to examine whether ARID1, as a transcription factor, binds *DUO1* directly. We tested ARID1 occupancy at the genomic region of *DUO1*, including the ∼1.4 kb promoter region upstream of the ATG and the ∼300 bp UTR region downstream of the stop codon in ARID1-GFP transgenic plants (wild type plants as the negative control), using an antibody against GFP. We sub-divided the genomic region of *DUO1* into ten subfragments of around 300 bp each (Figure 3A) and used *EIF4A1* as a negative control. An obvious ARID1 occupancy over most of the *DUO1* genomic region was detected in ARID1-GFP transgenic plants, with the peak of enrichment located between the ∼600–300 bp promoter region upstream of the ATG (Figure 3B, upper panel). In contrast, there was not much difference between wild type and ARID1-GFP plants for *EIF4A1* enrichment (Figure 3B, upper panel), and enrichment from the no antibody control was negligible (Figure 3C, lower panel). To confirm the results from the ChIP assay, we performed an in vitro DNA binding assay. We expressed ARID1 with an in vitro transcription/translation system (Figure 3C, the band shown in the “input” lanes), and validated that ARID1 directly binds the *DUO1* promoter region (Figure 3C). Therefore, both in vitro and in vivo evidence showed that ARID1 directly binds the *DUO1* promoter. Together with the observation of reduced *DUO1* expression in the *arid1* mutant, we therefore conclude that ARID1 acts as an activator of *DUO1*, which is important for the initiation of the second mitosis for sperm cell formation.

### ARID1 Locates in Nuclear Bodies and Is Developmentally Regulated during Sperm Cell Formation

Microarray analyses and our RT-PCR results indicated that *ARID1* is pollen-specific. In order to substantiate the RT-PCR analyses, we constructed a promoter-reporter construct of *proARID1:NLS-GFP*. The GFP reporter was undetectable in root and shoot apical meristems but a weak GFP signal was detected in the vegetative nucleus in tricellular pollen (TP) (Figure S3A). Microarray analysis [26] indicated that *ARID1* was more highly expressed (11 fold) in sperm than in pollen. Several genes with >7X higher expression in sperm than in pollen have been shown to be sperm-specific [2], [5], [10], [30]. We had therefore anticipated that *ARID1* expression would be sperm-specific, so the *ARID1* promoter:GFP reporter results were unexpected. We then examined the subcellular localization of pARID1:ARID1-GFP or -RFP fusion proteins in pollen. Microscopic analysis showed that ARID1-GFP localized to a single body in mature pollen (MP) (Figure S3B, white arrowheads). We occasionally (1%, n>500) observed a second ARID1-GFP body (Figure S3B, red arrowheads). To confirm the location of the ARID1-GFP body, we crossed HTR10-RFP, a sperm-specific marker [2] into *ARID1-GFP* transgenic plants. The ARID1-GFP body did not co-localize with HTR10 (Figure S3C, lower panel), but did co-localize with the vegetative nucleus in DAPI-stained mature pollen (Figure S3C, upper panel). We therefore concluded that ARID1-GFP was only present in the vegetative nucleus in mature pollen.

**Figure 4:**
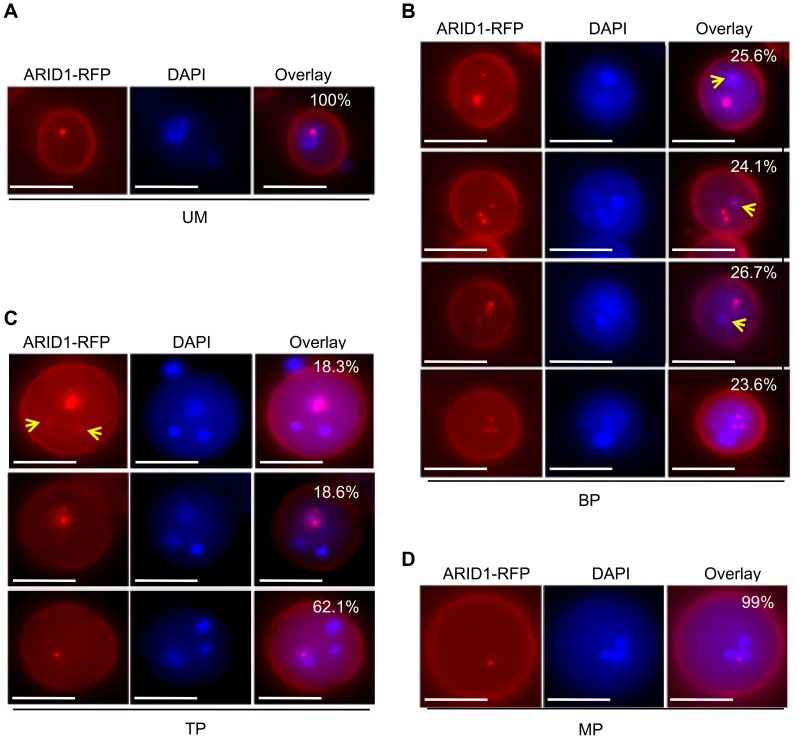
ARID1 localization is developmentally dynamic during sperm cell formation. Representative images of unicellular microspores (UM) (A), bicellular pollen (BP) (B), tricellular pollen (TP) (C), and mature pollen (MP) (D) from *ARID1-RFP* transgenic plants. Yellow arrows indicate RFP signals in the generative nuclei and sperm cells. The percentages indicate incidence of each pattern. 600–800 pollen grains were analyzed for each stage. Scale bar, 10 µm.

Considering that ARID1 can bind to the *DUO1* promoter directly to promote *DUO1* expression in both bicellular pollen and mature pollen (Figure 2, Figure 3, and Figure S2A), and *DUO1* is specifically expressed from the early bicellular stage to mature pollen [3], we hypothesized that ARID1 might overlap with DUO1, in a cell type-specific pattern. We therefore examined the localization of ARID1 nuclear bodies at different developmental stages in ARID1-GFP and ARID1-RFP transgenic plants. To avoid interference due to the partial overlap of excitation wavelengths for DAPI and GFP, we mainly used ARID1-RFP transgenic plants for these observations. As in mature pollen (Figure S3B and S3C), the ARID1-RFP body was a single nuclear body in unicellular microspores (UM) (Figure 4A). Unexpectedly, increased numbers of nuclear bodies were observed in bicellular pollen (Figure S4). We categorized the ARID1-RFP nuclear bodies in bicellular pollen (BP) into four distinct patterns with similar incidences (Figure 4B): all foci within the vegetative nucleus; multiple foci in both the vegetative nucleus and the generative nucleus; several foci in the vegetative nucleus but a single dot in the generative nucleus; and much larger foci in the vegetative nucleus but a less intense single dot in the generative nucleus (yellow arrows indicate signals in the generative cell). Additional representative examples are shown in Figure S4C. Although the overall signal intensity in vegetative nuclei was much stronger than that in the generative cell, these images show that ARID1-RFP was in both the vegetative cell and generative cell at the bicellular stage, which is obviously different from the distribution in UM and MP. In TP, three patterns of ARID1-RFP bodies were identified (Figure 4C): most pollen (>60%) had a single ARID1-RFP body, as in mature pollen, but the rest had multiple foci in the vegetative nucleus or a single weakly fluorescent ARID1-RFP body in sperm nuclei (yellow arrows). Only a single nuclear body in the vegetative nucleus was observed in mature pollen (Figure S3B, S3C, and MP, Figure 4D). To further substantiate the biological significance of the presence of ARID1 in the generative cell, we constructed transgenic plants with *ARID1* driven by *LAT52* (LAT52:ARID1), a vegetative cell-specific promoter, and *HTR10* (HTR10:ARID1), a generative cell and sperm cell-specific promoter, respectively. We observed reduced seed set (Figure S5A) and increased *DUO1* expression (Figure S5B) in >10 independent T1 HTR10:ARID1 plants but not in LAT52:ARID1 plants, indicating that accumulation of ARID1 in the generative cell is biologically relevant during pollen development. Taken together, these data indicate that the subcellular distribution of ARID1-RFP nuclear bodies is variable during pollen development. The transient localization of ARID1-RFP in the generative nucleus is consistent with the idea that ARID1 might be required for initiation or progression of the second mitosis, by promoting *DUO1* activation.

### ARID1 Interacts with Histone Deacetylase 8

**Figure 5:**
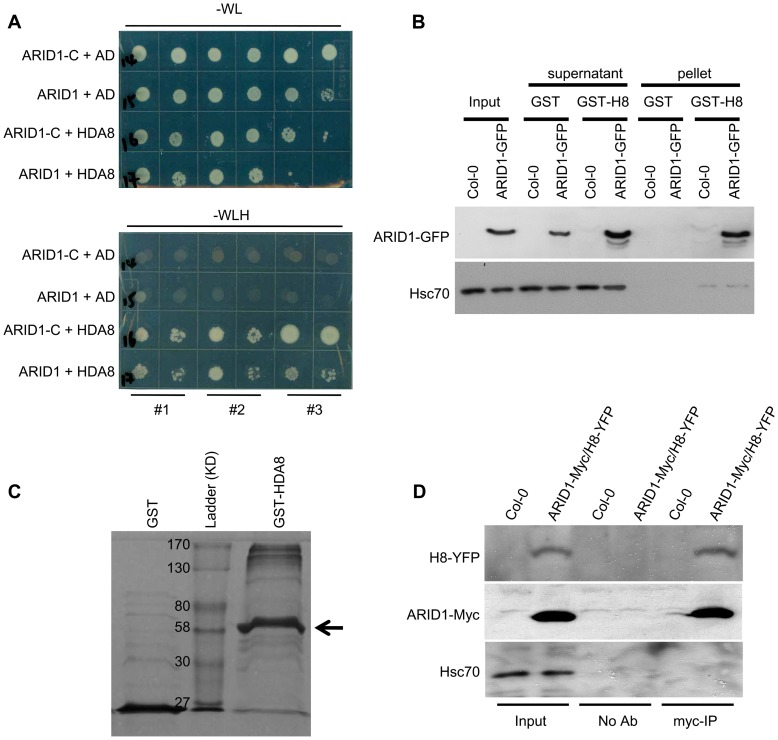
ARID1 physically associates with Histone Deacetylase 8 (HDA8). (A) Yeast cells co-expressing the indicated plasmids were grown on control (without Tryptophan and Leucine) or high stringency selection (without Tryptophan, Leucine, Histidine, and with 10 mM 3-AT) medium. *ARID1*, full length cDNA of *ARID1*; *ARID1-C*, the C-terminus-containing ELM2 domain of ARID1; *HDA8*, full length cDNA of *HDA8*. AD is pGAD10. The cultures from each of the indicated strains were diluted 100-fold and spotted. Three colonies were streaked for each pair. (B) ARID1 interacts with HDA8 by GST pulldown assay. Whole cell extracts from wild type or ARID1-GFP plants were applied onto GST and GST-HDA8 (abbreviated GST-H8) beads. GFP and Hsc70 (loading control) antibodies were used in immunoblotting. (C) Stained protein gel showing proteins used for the GST pulldown assay. 1/30 of the amount of each protein used in Figure 5A was resolved by SDS-PAGE. GST-H8, full length HDA8 fused to GST; GST alone was used as a control. GST-H8 is marked with an arrowhead. (D) ARID1 interacts with HDA8 by Co-IP. Inflorescences from ARID1-Myc; HDA8-YFP (abbreviated H8-YFP) doubly transgenic plants or from wild type were immunoprecipitated with Myc antibody. Myc, GFP and Hsc70 (loading control) antibodies were used for immunoblotting.

A human ELM2 domain-containing protein, MI-ER, directly interacts with Histone Deacetylase 1 (HDAC1) [27], [28]. To determine whether ARID1 might be associated with plant histone deacetylase complexes, we performed yeast two hybrid experiments. We detected an interaction (Figure 5A) between ARID1 and HDA8 (Histone Deacetylase 8), which is highly expressed in the vegetative nucleus but not in the generative nucleus or sperm cell nuclei (Figure S6A). Moreover, we found that the ELM2 domain of ARID1 was important for the interaction with HDA8 (Figure 5A), as was shown for an ELM2 protein with a histone deacetylase in human cells [28]. To confirm the yeast results, a recombinant HDA8-GST protein (Figure 5C) was used for pulldown assays, with GST as a negative control. ARID1-GFP was pulled down by HDA8-GST but not by GST (Figure 5B). To confirm this association in vivo, we performed Co-IP experiments and showed that HDA8-YFP was co-immunoprecipitated with antibodies that recognize the ARID1-Myc fusion protein (Figure 5D).

**Figure 6:**
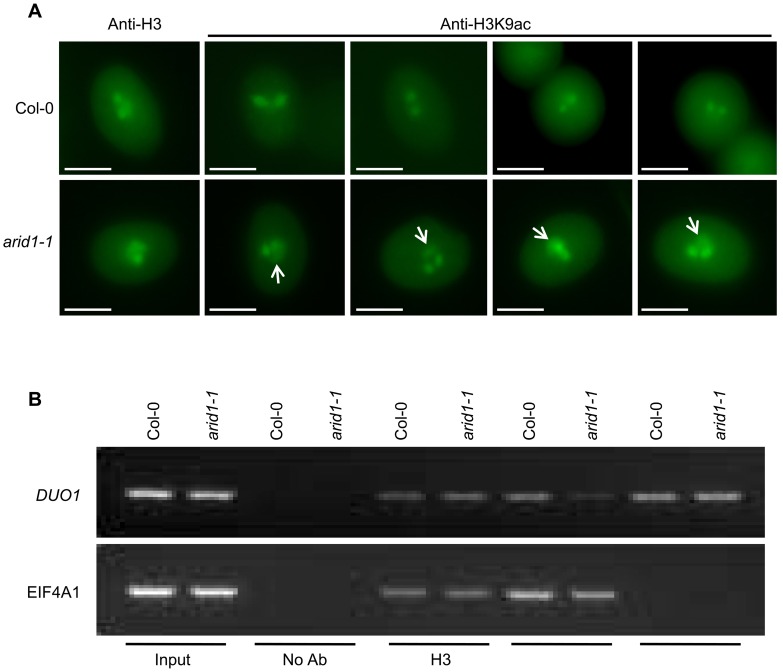
Altered histone acetylation in *arid1-1*. (A) Expanded pattern of histone acetylation signal in *arid1-1* by immunofluorescence analysis. Mature pollen from wild type and *arid1-1* plants was incubated with anti-H3 (control) and anti-H3K9ac antibodies. Representative examples for *arid1-1* show histone acetylation in the vegetative nucleus (indicated by white arrowheads). 50–100 pollen grains for each genotype were examined. (B) Reduced histone acetylation at the *DUO1* promoter in the *arid1-1* mutant by ChIP. Inflorescences from wild type and *arid1-1* were used for a ChIP assay with anti-H3, anti-H3K9ac, and anti-H3K4me3 antibodies. ChIP-DNA was used for PCR by amplifying 35 cycles for both *EIF4A1* (negative control) and the *DUO1_3* fragment, which bound ARID1. Similar results were obtained from three independent biological replicates; the results shown are from one replicate.

Since ARID1 interacts with HDA8, we predicted that the in vivo histone acetylation level might be altered in the *arid1-1* mutant. We therefore performed immunofluorescence with antibodies specific to H3K9 acetylation. In wild type pollen, the signal was only detected in the two sperm nuclei (Figure 6A, upper panel), but in the *arid1-1* mutant, the immunofluorescence signal was also detected in the vegetative nucleus (Figure 6A, lower panel). The immunofluorescence signal with the Histone 3 antibody, used as a control, showed no difference between wild type and *arid1-1* (Figure 6A). These results indicate that ARID1 is required to restrict histone acetylation to sperm cells.

For genes encoding proteins, increased expression is usually accompanied with increased active marks in euchromatin, that is histone H3 lysine 9 acetylation (H3K9Ac) and histone H3 lysine 4 trimethylation (H3K4me3). Antibodies to a constitutive histone (H3) reacted similarly with the control gene *EIF4A1* and with *DUO1*, in both wild type and the *arid1* mutant (Figure 6B). However, in the *arid1* mutant the H3K9Ac level was obviously reduced at *DUO1* but not at *EIF4A1* (Figure 6B). Furthermore, in the *arid1* mutant there was no detectable difference in the level of H3K4me3 at *DUO1* or *EIF4A1* (Figure 6B), suggesting that it is specifically histone acetylation that contributes to the reduced *DUO1* expression. In addition, we detected slight de-repression of transposable elements (TEs) in the *arid1-1* mutant (Figure S6B), accompanied with increased histone acetylation at these loci (Figure S6C), indicating that ARID1-mediated histone deacetylation activity also contributes to the silencing of TEs.

## Discussion

Sperm cell formation is regulated by several genes [31]. Among these genes, *DUO1* plays a key role in the initiation of the second mitosis and acts as a switch to turn on the expression of other germline-related genes [9]. No positive element has been reported to regulate *DUO1* expression but, in addition to the negative regulation mediated by miR159 [12], a putative repressive GRSF (Germline-Restrictive Silencing Factor) binding site was noted in the *DUO1* promoter [32]. However, mutagenesis of the putative GRSF binding site did not affect germline-specific expression of *DUO1* [9] and the ∼150 bp proximal *DUO1* promoter sequences that excluded the putative GRSF binding site were sufficient for germline-specific expression [9]. These results suggest that activation of the *DUO1* promoter may depend on transcription factors that bind to the proximal region of the promoter and that are inherited and/or segregated during asymmetric division of the microspore.

Here we provide evidence for such a positive transcription factor, ARID1. ChIP analysis and a DNA binding assay showed that ARID1 directly binds to the ∼300–600 bp promoter region adjacent but distal to the 150 bp proximal region of the promoter (Figure 3). We surmise that the discrepancy from the observations in the previous study was based on whether or not the expression driven by the 150 bp proximal promoter was sperm cell-specific [9], but ignored the difference in expression level in the generative cell between the intact promoter and the 150 bp proximal promoter. Thus we suggest that ARID1 binding to the *DUO1* promoter facilitates the activation of *DUO1*. To support the biological significance of this binding, we showed that disruption of *ARID1* resulted in reduced *DUO1* expression in germline cells (Figure 2). Although we did not observe decreased expression of three *DUO1* direct targets (*HTR10*, *GCS1*, and *GEX2*), DUO1-mediated CYCB1 accumulation in the generative cell was affected in the *arid1* mutant (Figure 2C). The unaffected expression of *GCS1* and *GEX2* in the *arid1* mutant might be explained by redundancy with DUO3, since it also promotes expression of *GCS1* and *GEX2*, but not of *CYCB1* [7], and the expression of *DUO3* was not affected in *arid1* (Figure 2A). Together with the normal sperm cell formation in *htr10*, possibly due to redundancy with other *HTR* members, we suggest that defective sperm cell formation in *arid1* results only from the disrupted function of the DUO1-CYCB1 module in germ cell division and not the function of DUO1-GCS1/GEX2 in germ cell specification, both of which contribute to severely defective sperm cell formation in *duo1*. Furthermore, that only the DUO1-CYCB1 module was disrupted, and not the DUO1-GCS1/GEX2/HTR10 module, possibly explains the much weaker phenotype in *arid1-1*, since specification of germ cells in *arid1-1* might be maintained by the remnant DUO1 and/or DUO3-mediated activation of GCS1/GEX2/HTR10. We presume that DUO1-CYCB1-mediated generative cell division is prerequisite for sperm cell formation, and that unaffected DUO3 should partially suppress the phenotype of single sperm cell-like pollen in *arid1*, if DUO1 and/or DUO3-GCS1/GEX2/HTR10-mediated germ cell specification is parallel with DUO1-CYCB1-mediated germ cell division.

**Figure 7:**
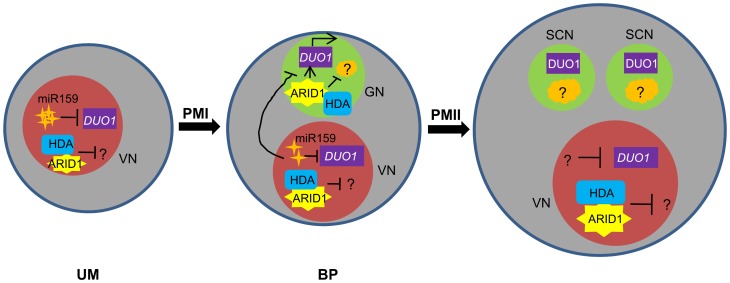
Model for ARID1 function during sperm cell formation in *Arabidopsis*. miR159 plays a major role in restricting *DUO1* expression in the vegetative cell. As pollen development proceeds, miR159 abundance is gradually decreased and ARID1 expands its expression into the generative cell, possibly by responding to the decreased repressive role of miR159 in bicellular pollen. ARID1 then promotes *DUO1* activation by directly binding to the *DUO1* promoter, and thereby facilitates the initiation of sperm cell formation. On the other hand, ARID1 might repress expression of unknown negative regulators (orange) of cell cycle progression, by altering the epigenetic status of the generative cell. Once sperm cells are formed, ARID1 gradually decreases until it is eliminated from germline cells, so that DUO1 is steadily present and negative regulators possibly related to germline function start to be active, due to dissociation of ARID1 and histone deacetylase. Thus we hypothesize that the alteration of epigenetic status during sperm cell formation is correlated with a change in the subcellular localization of ARID1, which could facilitate cell cycle progression of the two consecutive mitoses. VN, vegetative nucleus; GN, generative cell; SC, sperm cells.

In addition, unlike the *DUO1* expression pattern, ARID1 is initially expressed in the microspore nucleus and subsequently expanded or segregated into the generative nucleus during the first asymmetric division (Figure 4 and Figure S4), indicating that *DUO1* activation is an active process mediated by ARID1 from the vegetative cell, and not passively accomplished due to the completion of the first asymmetric division. We propose a model (Figure 7) to explain how *DUO1* could be coordinately and sequentially regulated by the negative regulator miR159 and by the positive regulator ARID1. In the vegetative cell, *MIR159* is transcribed abundantly during the unicellular stage, and so might play a major role in blocking *DUO1* expression. As pollen development proceeds, in spite of the gradually decreasing but still detectable repressive role of *miR159* in bicellular pollen, ARID1, inherited from the microspore, partitions into the generative cell to bind to *DUO1* and gradually promote *DUO1* activation. We hypothesize that other factors together with AIRD1 are potentially involved in *DUO1* activation, as *duo1* is 100% penetrant and because the generative cell fails to divide in all *duo1* pollen grains. In contrast, disruption of ARID1 only caused partial defects in sperm cell formation. Due to the absence of DUO1 accumulation in the vegetative nucleus of *arid1-1*, we hypothesize that unknown factors (other than ARID1-associated histone modification machinery) might take over the major role of miR159 restricting *DUO1* expression in the vegetative cell nucleus in the weak *arid1-1* mutant (Figure 2). In parallel, ARID1 might promote sperm cell formation by altering the epigenetic status in both the vegetative cell and the generative cell; ARID1 physically associates with histone deacetylases, which could affect expression of the unknown gene(s) involved in sperm cell formation. Our data showed that ARID1 is necessary for the balance of histone acetylation between the vegetative cell and sperm cells of mature pollen (Figure 6A), indicating that this characteristic could be carried over from bicellular pollen. Moreover, a recent study showed that increased histone acetylation in cultured microspores led to the switch from gametophytic division to sporophytic division [33], further indicating that histone acetylation is critical for cell cycle progression during sperm cell formation.

Given the permanent presence of ARID1 in the vegetative cell and its short stay in the germline cell (Figure 4), we speculate that ARID1 might carry information that strengthens communication between two distinct cell types, facilitating initiation of the second mitosis or the process of sperm cell formation, by acting on related genes, in addition to the key regulator, DUO1. Our results indicated that ARID1-mediated *DUO1* activation is important for sperm cell formation. It will be interesting to discover whether ARID1 regulates other genes and more broadly to deduce the functions of other plant ARID proteins, in order to understand the role of the conserved ARID domain. In animals, ARID proteins have been implicated in a variety of biological processes including embryonic development, cell lineage, gene regulation and cell cycle control. ARID1 is perhaps analogous to ARID4A and ARID4B in animals, which associate with the histone deacetylase complex [16],[17] and are involved in male fertility control by acting in the Retinoblastoma (RB) pathway [15]. ARID4A is a RB-binding protein, and it is well established that the RB pathway controls cell cycle progression in a variety of organisms [34], including plants [35], [36]. A plant homologue of RB, RBR (Rb-related), plays a pivotal role in male gametophyte patterning by regulating cell division and cell fate [37], as both the vegetative cell and generative cell over-proliferated in *rbr* mutants [37]. As this phenotype was not seen in the *arid1* mutant or in *arid1* antisense plants and as ARID1 does not contain a pRB-binding motif, LXCXE [16], it is unlikely that ARID1 binds RBR. However, there are 10 ARID proteins in *Arabidopsis* [24]and several other *ARID*s are expressed in pollen [26], [38], so it is possible that other ARIDs are involved in the RB pathway to regulate sperm cell formation.

In addition to its transient presence in the germline cell, ARID1 is in the vegetative nucleus from the microspore to mature pollen stage (Figure 4). What does ARID1 do in the vegetative cell? There are two possibilities. First, ARID1, as a transcription factor, might regulate expression of unknown genes in the vegetative cell. This regulation perhaps includes transcriptional activation, such as for *DUO1*, and transcriptional repression. Together with the association of ARID1 with histone modification machinery, we suggest that ARID1 is required to regulate the chromatin environment around *DUO1* so that it can achieve its maximal/optimal expression. Second, ARID1 in the vegetative cell might be required for maintaining the level of transposable element (TE) de-repression, as de-repression of many TEs occurs in the vegetative cell [39], although the biological significance of TE de-repression remains unclear. We demonstrated greater TE de-repression in *arid1-1* pollen (Figure S6B), which was accompanied with increased histone acetylation at those de-repressed TE loci (Figure S6C). Therefore, we propose that the function of ARID1 in the vegetative nucleus is to modulate the overt de-repression of TEs by association with the histone modification machinery. A dual role for ARIDs, either as transcriptional activators or transcriptional repressors, has been reported in animals [21], [23]. Moreover, the ARID1 nuclear body (Figure 4) might be a processing center for ARID1-mediated histone modifications. We showed [40] that Cajal bodies, which are processing centers for RNA-directed DNA Methylation (RdDM), were similarly developmentally variable during sperm formation

## Materials and Methods

### Plant Materials

The *arid1-1* T-DNA insertion mutant (SALK_047099) was obtained from the ABRC (www.arabidopsis.org). Seeds of HTR10-RFP and DUO1-RFP were kindly provided by Fred Berger and David Twell, respectively.

### Plasmid Construction

For the construction of ARID1-GFP, ARID1-RFP, and ARID1-Myc plasmids, *ARID1* was amplified from wild type (Columbia-0) genomic DNA with the primer pair ARID1F1 and ARIDR1, cloned into pENTR-D/TOPO, and then transferred into the plant expression destination vector pMDC107 to construct ARID1-GFP, into pMDC163 (GUS was replaced by a mRFP fusion tag) to construct ARID1-RFP, and into pEarleyGate303 to construct ARID1-Myc. For *ARID1* promoter analysis, a 1.5-kb fragment upstream of the ATG was amplified using the primer pair ARID1F1/R4 and was subcloned into pENTR-D/TOPO and then transferred into the plant expression vector pGII-NLS3XGFP. For constructing the antisense *ARID1* plasmid, cDNA was obtained using the primer pair ARID1F5/R5, cloned into pENTR-D/TOPO, then transferred into the plant expression vector pB7WG2 (digested with SacI and SpeI to replace the 35S promoter with the native *ARID1* promoter and then digested with KpnI and ApaI to insert the LAT52-GFP cassette). To construct the LAT52:ARID1 and HTR10:ARID1 plasmids, cDNA was obtained using the primer pair ARID1F6/R1, cloned into pENTR-D/TOPO, then transferred into the plant expression vector pB7WG2 (digested with SacI and SpeI to replace the 35S promoter with the LAT52 promoter or the HTR10 promoter, respectively, then digested with KpnI and ApaI to insert the LAT52-GFP cassette). To construct *ARID1* for the in vitro transcription and translation system, we inserted *ARID1* full length cDNA, amplified with the primer pairs ARID1F2/R6 into the pCMVTNT vector (Promega). For yeast two hybrid experiments, cDNAs of *ARID1* and *ARID1-C* were amplified from pollen RNA using RT-PCR and the primer pairs ARID1F2/R2 and ARID1F4/R2, then cloned into the pGBT9 yeast expression vector; the cDNA of *HDA8* was amplified using the primer pair HDA8F1/R1, then cloned into the pGAD10 yeast expression vector. For GST pulldown experiments, the cDNA of *HDA8* was amplified using primers HDA8F1/R1 and cloned into the pGEX-2TK vector. For the HDA8-YFP construct, *HDA8* was amplified from wild type genomic DNA with the primer pair HDA8F2/R2, cloned into pENTR-D/TOPO, and then transferred into the plant expression destination vector pGWB40. Primer sequences are listed in Table S1.

### Yeast-Two-Hybrid Experiments

The yeast two hybrid assays were performed according to the protocol available on the Clontech website, using strain AH109. Yeast transformation was performed using yeast transformation buffer (0.1 M LiAc, 40% PEG3350 in TE). Transformants were plated and selected on synthetic complete medium that lacked the specified amino acids. Positive colonies were inoculated and spotted with a 100-fold dilution onto synthetic complete medium lacking leucine, tryptophan, and histidine and containing 10 mM 3-amino-1,2,4-triazole. The plates were incubated for 2–4 d at 28°C for interaction analysis.

### ChIP

ChIP was performed according to [8]. ARID1 occupancy at the *DUO1* genomic region was determined by ChIP using a GFP antibody (Cat. 632460, Clontech, 1∶200) and inflorescences from wild type and ARID1-GFP transgenic plants, respectively. The occupancy of histone modification marks at *DUO1* was determined by ChIP using Histone 3 antibody (Cat.06-755, Upstate, 1∶50), Histone Lysine 9 acetylation antibody (Cat. 17-10241, Upstate, 1∶200), and Histone Lysine 4 trimethylation antibody (Cat.ab8580, abCam, 1∶200) and inflorescences from wild type and the *arid1-1* mutant, respectively. DNA present in the immunoprecipitates was quantified by qPCR or PCR relative to total input DNA. The results shown were consistent in two biological replicates. The primer sets used for the PCR are listed in Table S1.

### DNA Binding Assays

DNA binding assays were performed as described [41] with the following modifications. Briefly, biotinylated DNA fragments corresponding to 3, 4, 5, and 10 (Figure 3) were generated by PCR using primer pairs DUO1_F3/R3, DUO1_F4/R4, DUO1_F5/R5, and DUO1_F10/R10, with labeling by 5′biotin at F3, F4, F5, and F10, respectively. Then 100 pmol of the biotinylated DNA fragments were incubated with 50 ul prewashed Streptavidin Agoraose Resin (Thermo, Cat.20349) in IP100 buffer (100 mM potassium glutamate, 50 mM Tris-HCl pH 7.6, 2 mM MgCl2, 0.5% NP40) for 2 h at room temperature with slight rotation, and washed five times in IP100 buffer. In parallel, ARID1 was subjected to TNT T7 in vitro transcription/translation with the TNT Coupled Wheat Germ Extract System (Promega, Cat.L4140). 25 ul freshly translated ARID1 protein was added to DNA-bound beads in the IP buffer plus complete protease inhibitor cocktail, and the mixture was rotated at 4°C for 2 h. Beads were washed eight times with IP100 buffer, then proteins were stripped off the beads by boiling with 2XSDS buffer and then subjected to SDS-PAGE. The ARID1 protein bound by biotinylated DNA was detected by immunoblotting with a 1∶200 dilution of anti-ARID1 (The peptide “SMVADEDAVDYSKT” was conjugated to KLH and used to raise rabbit polyclonal antibodies (GL Biochem)).

### GST Pulldown

The pulldown assay was carried out as described previously [42]. GST and GST-HDA8 were expressed in *E. coli* BL21. Cells were disrupted by sonication and the proteins were purified by glutathione Sepharose 4B affinity chromatography. 600 µl of protein extract from inflorescences of ARID1-GFP plants was applied to the beads-protein mixture (30 µg total protein) and incubated for 2 h at 4°C on a rotating wheel. The beads were washed 5 times with IP lysis buffer. The bound (pellet) and unbound (supernatant) proteins were detected by immunoblotting with anti-GFP (Cat.632480, Clontech, 1∶2000 dilution) and anti-Hsc70 (Cat.SPA-818, Stressgen, 1∶10000 dilution) antibodies. 1/6 of the pellet fractions, and 1% and 0.1% of the supernatant fractions for anti-GFP and anti-Hsc70 IPs were used, respectively.

### Co-Immunoprecipitation

The immunoprecipitation assay was carried out as described [42]. One gram of inflorescences from wild type or ARID1-Myc; HDA8-YFP doubly transgenic plants were ground in liquid nitrogen and homogenized in 2 ml of protein lysis buffer (50 mM Tris–HCl at pH 7.5, 150 mM NaCl, 0.2% NP-40, 2 mM DTT, 10% glycerol, complete protease inhibitor cocktail). The lysates were incubated at 4°C for 50 min on a rotating wheel and centrifuged at 16000 g to pellet debris, and then supernatants were pre-cleared for 20 min with protein G agarose beads. Equivalent lysate was mixed with Myc antibodies (Cat. SC-70463, Santa Cruz, 1∶200) pre-coupled to protein G agarose beads or to beads alone, respectively. After incubation for 2 h at 4°C, the immune complexes were washed with lysis buffer. Proteins from 1/6 of the “No Ab” (no antibody) IP and 1/6 of the anti-myc IP were analyzed by immunoblotting using anti-Myc, anti-GFP, and anti-Hsc70. Proteins from 1/100 of the input were used for the anti-Myc and anti-GFP blots, while proteins from 1/1000 of the input were used for the anti-Hsc70 blot.

### Immunofluorescence Assay

Inflorescences were fixed for 30 minutes in methanol∶acetone (4∶1, v/v) at room temperature, and anthers were dissected to release tricellular pollen onto a slide covered with liquid pollen germination medium [43]. The slides were allowed to dry for about 20 min at room temperature and then were covered with a thin layer of agarose/gelatin/sucrose (0.94% low melting agarose/0.84% gelatin/0.3% w/v sucrose) for 10 minutes at 37°C. The slides were soaked in blocking buffer (PBS with 5% BSA) for 1 hour at 37°C, and then incubated with H3 antibody (1∶50 dilution, Cat. 06-755, Upstate) or H3K9ac antibody (1∶200 dilution, Cat. 1710241, Upstate), respectively, overnight at 4°C in a dark moist chamber. After washed with blocking buffer, pollen was incubated in blocking buffer containing Alexa Fluor 488 goat anti-rabbit antibody (1∶200 dilution, Cat.711-545-152, Jackson) for 6 h at room temperature. Slides were washed in PBS for five times, and observed with an Axiovert microscope under the GFP channel. Images were acquired using an AxioCamRM camera and AxioVision 4.8.1 software and processed using Adobe Photoshop CS2 (Adobe).

## Supporting Information

Figure S1 Characterization of *arid1-1* and phenotypic analysis of *arid1* pollen. (A) T-DNA insertion site and expression of *ARID1* in *arid1-1*. ARID and ELM2 denote the regions that encode those protein domains (upper panel). RT-PCR analysis of *ARID1* in *arid1-1* (lower panel). *UBQ5* was the loading control, the RT (−) control PCR was performed with *UBQ5* primers. P1, P2, P3 and P4 represent primers listed in Table S1. (B) Representative in vitro pollen germination assay with WT and *arid1-1*. (C) Representative images of WT and *arid1-1* in vivo pollen tube growth, as assessed by ovule clearing and Decolorized Aaniline blue staining. (D) Representative images of mature pollen from WT, *arid1-1*, and antisense *ARID1* transgenic plants (line#41) by DAPI staining. Red arrows indicate single-sperm-like pollen.
(TIF)

Figure S2 Expression of genes involved in sperm cell formation in the *arid1* mutant. (A) Expression of *DUO1* in pollen of *arid1-1* and antisense lines by RT-PCR. Line #24 is a strong line with severely reduced seed set, Line #41 is a moderate line with moderately reduced seed set, and Lines #26, #42, #43, #48 are weak lines with slightly reduced seed set. The RT (−) control PCR was performed with *UBQ5* primers. Results from one of two biological replicates are shown. (B) Expression of *MIR159a*, *MIR159b*, and *MIR159c* in pollen from WT (light grey) and *arid1-1* (dark grey). *UBIQUITIN5* (*UBQ5*) was the loading control. All measurements represent the average of three biological replicates with error bars representing the standard error of the mean (SEM). (C) APC8 was not affected in the *arid1* mutant. Transgenic plants with APC8-YFP (a protein fusion) were crossed with *arid1-1*. Scale bar, 10 µm. (D) ARID1 is not required for the expression of *GEX1*, *GEX2*, and HTR10. Transgenic plants with *pGEX1-GFP* (a promoter fusion), *pGEX2-GFP* (a promoter fusion), and HTR10-RFP (a protein fusion) were crossed with *arid1-1*. Scale bar, 10 µm.
(TIF)

Figure S3 ARID1 is located in the vegetative nucleus of mature pollen. (A) Representative images of microspores or pollen from plants harboring the *proARID1:NLS-GFP*. Scale bar, 10 µm. (B) Representative images of mature pollen from two independent transgenic plants harboring the *proARID1:ARID1-GFP* construct. White arrowheads and red arrowheads indicate single dot and twin dots, respectively. (C) Representative images showing the nuclear constitution in mature pollen of ARID1-GFP plants. The left panel for each represents GFP epifluorescence; the middle panel shows DAPI staining or the HTR10-RFP signal, respectively; the right panel shows an overlay of the left and middle panels. Scale bar, 10 µm.
(TIF)

Figure S4 ARID1 nuclear bodies are variable in bicellular pollen. Two representative fields showing multiple and variable ARID1 nuclear bodies in bicellular pollen. Both ARID1-GFP (A) and ARID1-RFP (B) driven by the native promoter were introduced into Col-0 plants. (C) is an enlarged view of additional ARID1-RFP bicellular pollen. The white arrows indicate signal in generative nuclei.
(TIF)

Figure S5 Overexpression of *ARID1* caused increased *DUO1* expression and reduced fertility. (A) Seed set analysis in LAT52:ARID1 and HTR10:ARID1 transgenic plants. Numbers represent two individual T1 lines from each construct, and the percentage of normal seeds in each line is shown. ∼10 siliques from the middle part of the primary shoot for each plant were analyzed. (B) Expression of *DUO1* and *ARID1* in mature pollen from Col-0 and from LAT52:ARID1 and HTR10:ARID transgenic plants. *UBIQUITIN5* (*UBQ5*) was the loading control. All measurements represent the average of two biological replicates with error bars representing the standard error of the mean (SEM).
(TIF)

Figure S6 De-repressed TEs and increased histone acetylation in the *arid1* mutant. (A) Representative images of microspores or pollen from plants harboring *HDA8-YFP*. (B) Expression of TEs in *arid1-1*. cDNAs from pollen of wild type (Col-0), *arid1-1*, *arid3-4* (a mutant of another *ARID*, unpublished), and *rdr2-1* (a siRNA biogenesis mutant as a positive control) were used as templates for PCR reactions with AtSN1, soloLTR, siR02, and AtGP1. All amplifications were for 35 cycles, except UBQ5, which was for 25 cycles. The red arrow indicates the specific band for AtGP1. (C) Increased histone acetylation in *arid1-1*. ChIP DNA samples obtained with H3K9ac antibodies or without antibody (No Ab) as a control were templates for PCR reactions of all tested loci. Amplifications were for 35 cycles.
(TIF)

Table S1 Primers used in this study.
(DOC)
