# Supplementary figures and images for: An ARID Domain-Containing Protein within Nuclear Bodies Is Required for Sperm Cell Formation in Arabidopsis thaliana
Source: PLoS Genet. 2014 Jul 24;10(7):e1004421. doi: 10.1371/journal.pgen.1004421 (PMC4109846; doi:10.1371/journal.pgen.1004421)

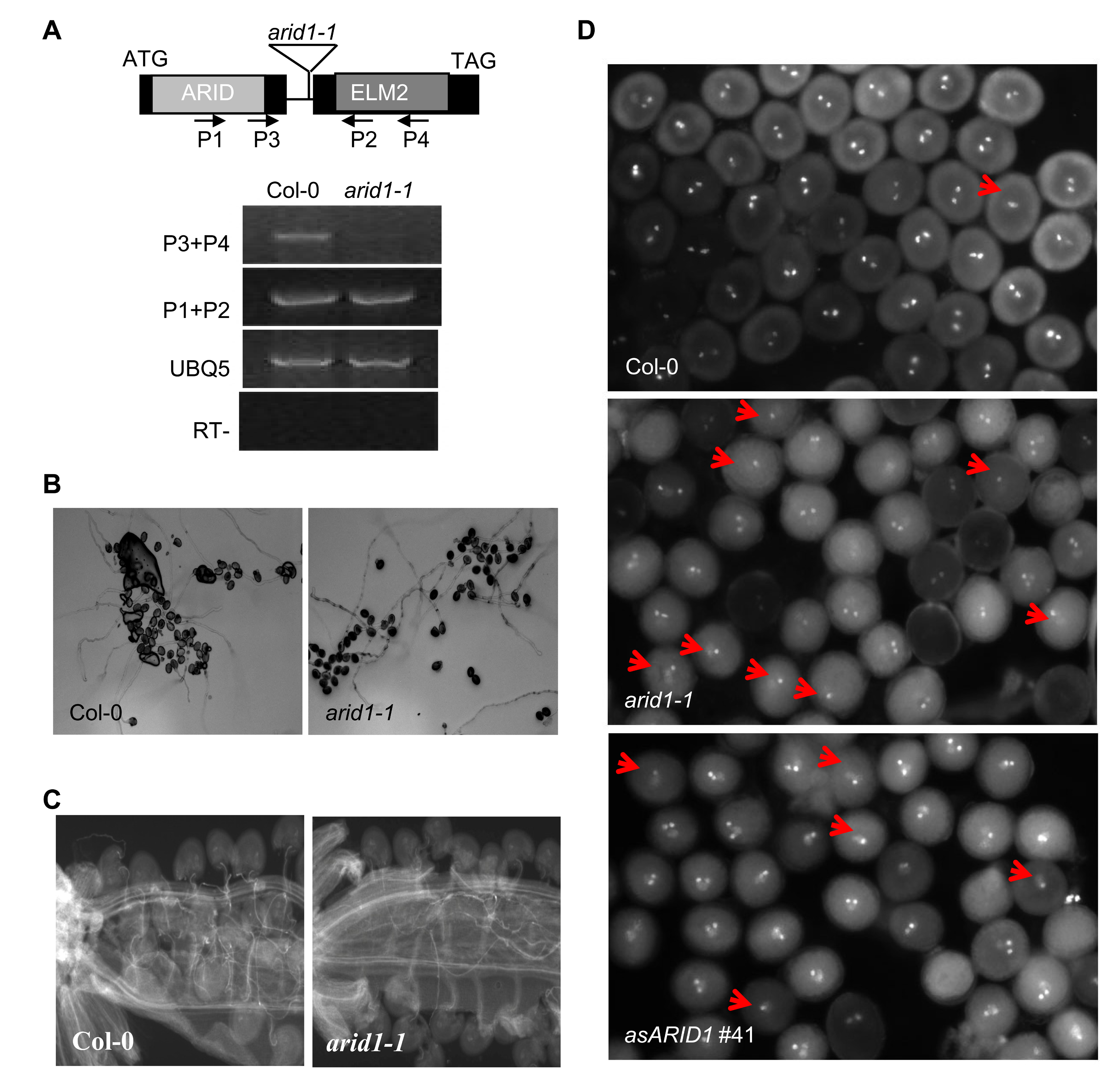

Supplement: Figure S1 — Characterization of arid1-1 and phenotypic analysis of arid1 pollen. (A) T-DNA insertion site and expression of ARID1 in arid1-1. ARID and ELM2 denote the regions that encode those protein domains (upper panel). RT-PCR analysis of ARID1 in arid1-1 (lower panel). UBQ5 was the loading control, the RT (−) control PCR was performed with UBQ5 primers. P1, P2, P3 and P4 represent primers listed in Table S1. (B) Representative in vitro pollen germination assay with WT and arid1-1. (C) Representative images of WT and arid1-1 in vivo pollen tube growth, as assessed by ovule clearing and Decolorized Aaniline blue staining. (D) Representative images of mature pollen from WT, arid1-1, and antisense ARID1 transgenic plants (line#41) by DAPI staining. Red arrows indicate single-sperm-like pollen. (TIF) [file pgen.1004421.s001.tif]

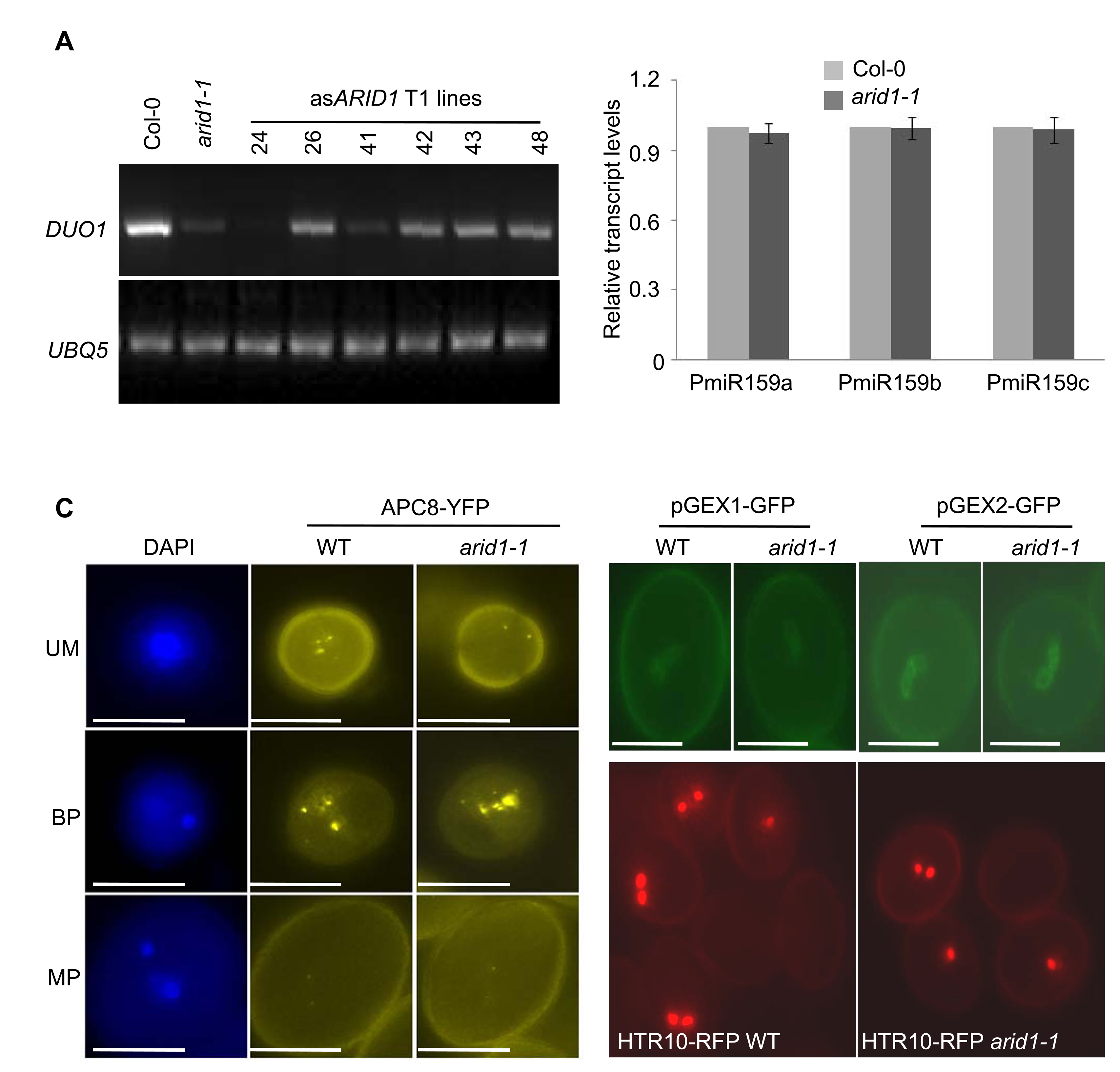

Supplement: Figure S2 — Expression of genes involved in sperm cell formation in the arid1 mutant. (A) Expression of DUO1 in pollen of arid1-1 and antisense lines by RT-PCR. Line #24 is a strong line with severely reduced seed set, Line #41 is a moderate line with moderately reduced seed set, and Lines #26, #42, #43, #48 are weak lines with slightly reduced seed set. The RT (−) control PCR was performed with UBQ5 primers. Results from one of two biological replicates are shown. (B) Expression of MIR159a, MIR159b, and MIR159c in pollen from WT (light grey) and arid1-1 (dark grey). UBIQUITIN5 (UBQ5) was the loading control. All measurements represent the average of three biological replicates with error bars representing the standard error of the mean (SEM). (C) APC8 was not affected in the arid1 mutant. Transgenic plants with APC8-YFP (a protein fusion) were crossed with arid1-1. Scale bar, 10 µm. (D) ARID1 is not required for the expression of GEX1, GEX2, and HTR10. Transgenic plants with pGEX1-GFP (a promoter fusion), pGEX2-GFP (a promoter fusion), and HTR10-RFP (a protein fusion) were crossed with arid1-1. Scale bar, 10 µm. (TIF) [file pgen.1004421.s002.tif]

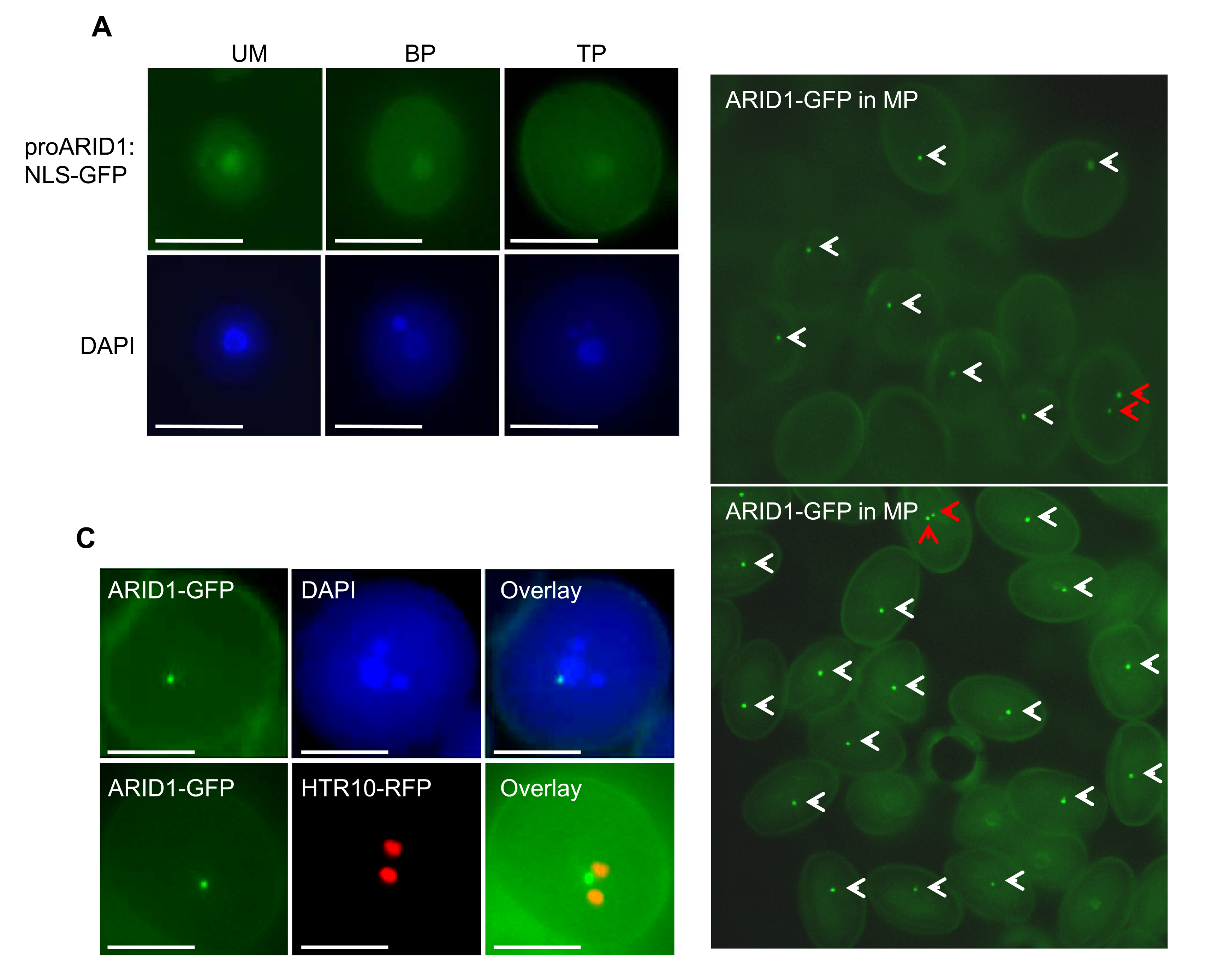

Supplement: Figure S3 — ARID1 is located in the vegetative nucleus of mature pollen. (A) Representative images of microspores or pollen from plants harboring the proARID1:NLS-GFP. Scale bar, 10 µm. (B) Representative images of mature pollen from two independent transgenic plants harboring the proARID1:ARID1-GFP construct. White arrowheads and red arrowheads indicate single dot and twin dots, respectively. (C) Representative images showing the nuclear constitution in mature pollen of ARID1-GFP plants. The left panel for each represents GFP epifluorescence; the middle panel shows DAPI staining or the HTR10-RFP signal, respectively; the right panel shows an overlay of the left and middle panels. Scale bar, 10 µm. (TIF) [file pgen.1004421.s003.tif]

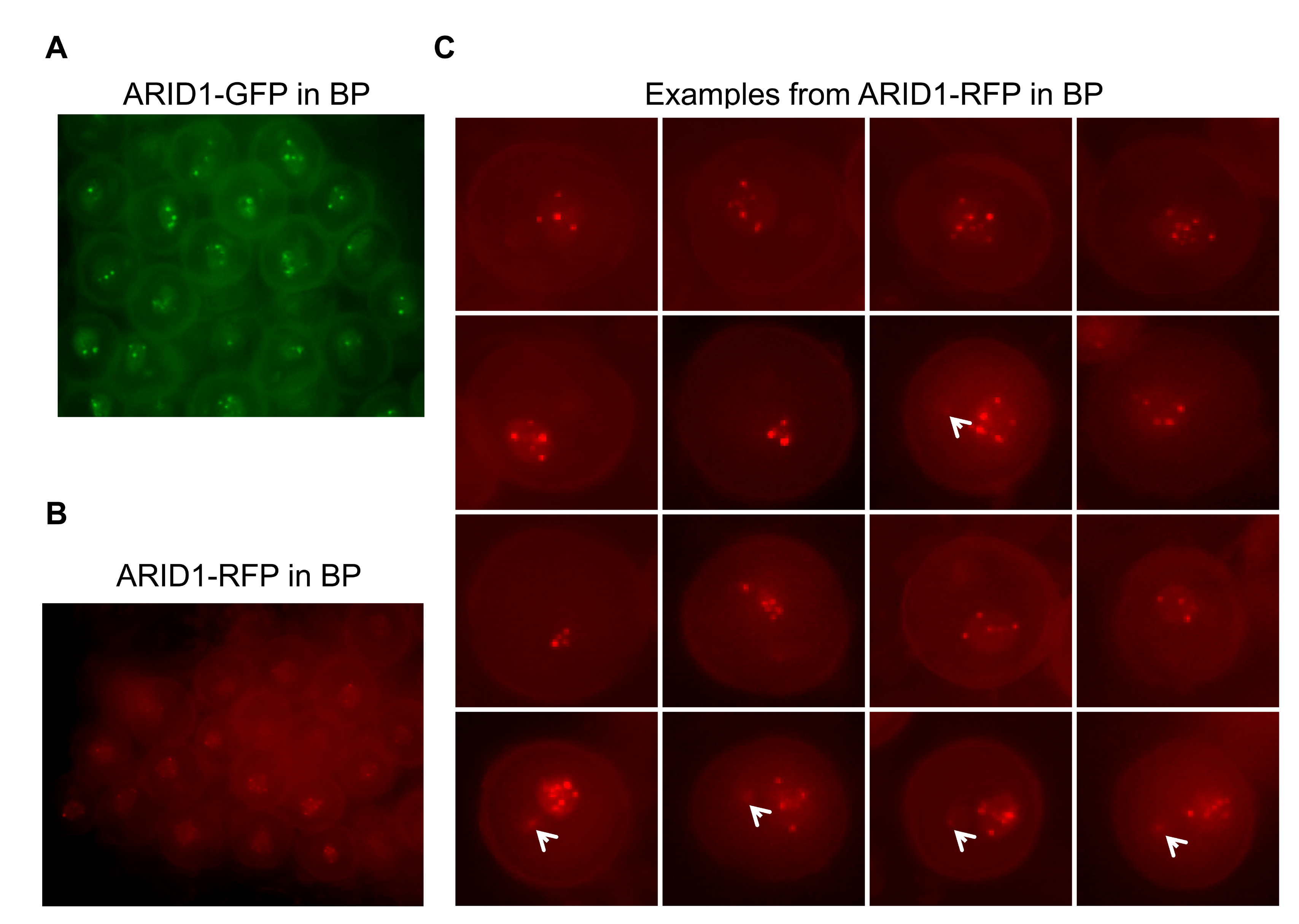

Supplement: Figure S4 — ARID1 nuclear bodies are variable in bicellular pollen. Two representative fields showing multiple and variable ARID1 nuclear bodies in bicellular pollen. Both ARID1-GFP (A) and ARID1-RFP (B) driven by the native promoter were introduced into Col-0 plants. (C) is an enlarged view of additional ARID1-RFP bicellular pollen. The white arrows indicate signal in generative nuclei. (TIF) [file pgen.1004421.s004.tif]

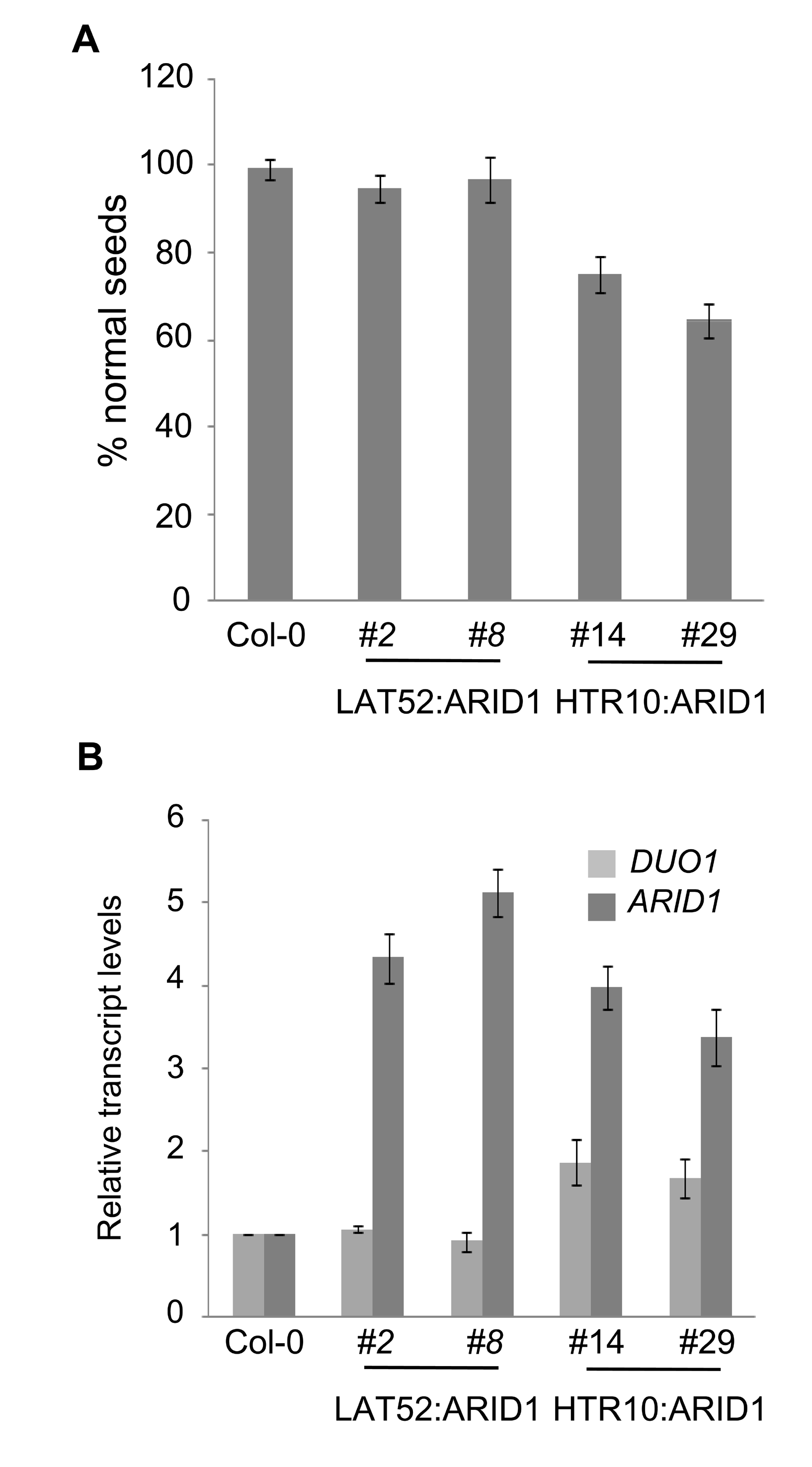

Supplement: Figure S5 — Overexpression of ARID1 caused increased DUO1 expression and reduced fertility. (A) Seed set analysis in LAT52:ARID1 and HTR10:ARID1 transgenic plants. Numbers represent two individual T1 lines from each construct, and the percentage of normal seeds in each line is shown. ∼10 siliques from the middle part of the primary shoot for each plant were analyzed. (B) Expression of DUO1 and ARID1 in mature pollen from Col-0 and from LAT52:ARID1 and HTR10:ARID transgenic plants. UBIQUITIN5 (UBQ5) was the loading control. All measurements represent the average of two biological replicates with error bars representing the standard error of the mean (SEM). (TIF) [file pgen.1004421.s005.tif]

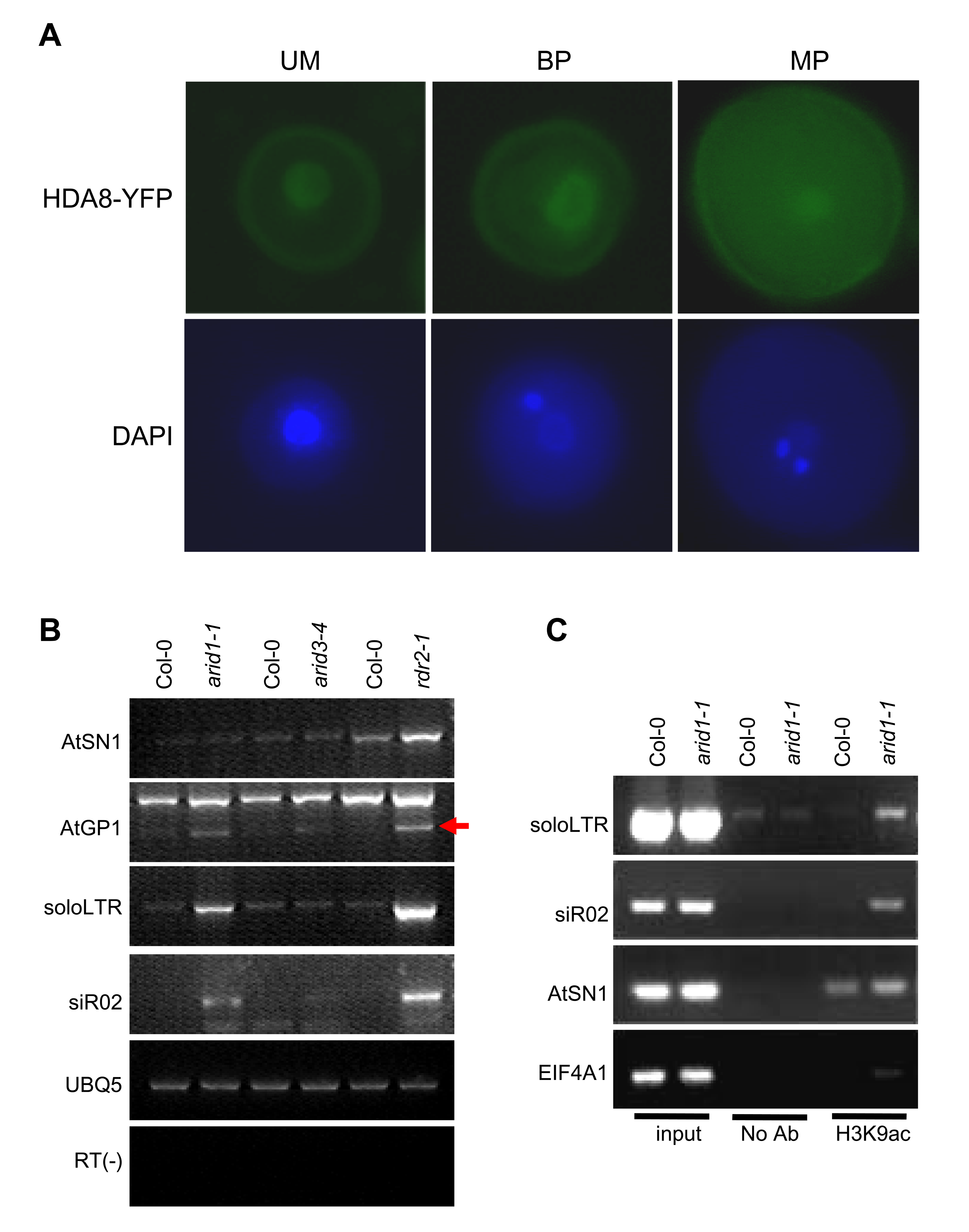

Supplement: Figure S6 — De-repressed TEs and increased histone acetylation in the arid1 mutant. (A) Representative images of microspores or pollen from plants harboring HDA8-YFP. (B) Expression of TEs in arid1-1. cDNAs from pollen of wild type (Col-0), arid1-1, arid3-4 (a mutant of another ARID, unpublished), and rdr2-1 (a siRNA biogenesis mutant as a positive control) were used as templates for PCR reactions with AtSN1, soloLTR, siR02, and AtGP1. All amplifications were for 35 cycles, except UBQ5, which was for 25 cycles. The red arrow indicates the specific band for AtGP1. (C) Increased histone acetylation in arid1-1. ChIP DNA samples obtained with H3K9ac antibodies or without antibody (No Ab) as a control were templates for PCR reactions of all tested loci. Amplifications were for 35 cycles. (TIF) [file pgen.1004421.s006.tif]
