# Supplementary material for: An ARID Domain-Containing Protein within Nuclear Bodies Is Required for Sperm Cell Formation in Arabidopsis thaliana
Source: PLoS Genet. 2014 Jul 24;10(7):e1004421. doi: 10.1371/journal.pgen.1004421 (PMC4109846; doi:10.1371/journal.pgen.1004421)
Supplement: Table S1 — Primers used in this study. (DOC) [file pgen.1004421.s007.doc]

**Supplemental Table**

**Table S1 Primers used in this study.**

| Primer name | Sequence | Use |
| --- | --- | --- |
| ARID1F1 | CACCAAGAGACGGCGTGGCGGGAA | gDNA |
| ARID1R1 | TCGACATCTTTTTTTATTAGGCG |  |
| ARID1F2 | GCGGAATTCATGGCGGGTTGGTCGATGGTAG | cDNA |
| ARID1R2 | GTCGACGGCTATCGACATCTTTTTTTATTA |  |
| ARID1F3 | AAAGTCCAGAAGATGCACCCA | qPCR |
| ARID1R3 | AGCCTTGGTTTGTTCTTTGG |  |
| ARID1R4 | TAACAACGAACTACGAACCCTAC | promoter |
| ARID1F4 | GCGGAATTCCCATGTGCTCTAGTTGGGTCAA | cDNA |
| ARID1F5 | CACCTCGACATCTTTTTTTATTAGGC | antisense |
| ARID1R5 | ATGGCGGGTTGGTCGATGGTAG |  |
| ARID1R6 | GGCCTCGAGTCGACATCTTTTTTTATTAGGCG | cDNA |
| ARID1F6 | CACCATGGCGGGTTGGTCGATGGTAG | cDNA |
| HDA8F1 | CGCGGATCCATGGTTACCAATCGCGTAGACG | cDNA |
| HDA8R1 | CGCGGATCCGTCAAGTTCCTCTGAGAAATGGA |  |
| HDA8F2 | CACCCTGAACACAATCTTACTAAGTT | gDNA |
| HDA8R2 | AGTTCCTCTGAGAAATGGAACA |  |
| DUO1_1F | TGTTCTGGAAGTTTGTTGTTG | ChIP |
| DUO1_1R | CTCGAACGACATGATATGGCT |  |
| DUO1_2F | GGTTTGTGGATTCGGAGCCAT | ChIP |
| DUO1_2R | TACTATTCACTTGTTTTCGAA |  |
| DUO1_3F | GGCGAAAACAAGTGAATAG | ChIP |
| DUO1_3R | TTGGTAAACCACTAAATGAC |  |
| DUO1_4F | GTGAGTGCATCTGCACATTAG | ChIP |
| DUO1_4R | TGTGAAGGATCTTCGAGACTC |  |
| DUO1_5F | GTTTCAACTTTCAAGGCTCTC | ChIP |
| DUO1_5R | CCTCATCGCTAATCGATCTCT |  |
| DUO1_6F | CGAGAGAGAGATCGATTAGCG | ChIP |
| DUO1_6R | GCATCCACTATAAACAAACAC |  |
| DUO1_7F | TGGATGCAAGTTCTCGGCTGA | ChIP |
| DUO1_7R | CAAAAAACCGAAAAACAGAGC |  |
| DUO1_8F | GATGATTTACAAAATTCACAA | ChIP |
| DUO1_8R | AAGGGTCGTCAAGTCTAGCCA |  |
| DUO1_9F | GCAGAGAGCTTTTGGCTAGAC | ChIP |
| DUO1_9R | CAAGAATTGTCATTACATAAG |  |
| DUO1_10F | GGTCTATAATCTAACTACATT | ChIP |
| DUO1_10R | TCAAATTGTCTTCAGTGTCCT |  |
| EIF4A1_F | TCTTGGTGAAGCGTGATGAG | ChIP |
| EIF4A1_R | GCTGAGTTGGGAGATCGAAG |  |
| DUO1F | CTGACGAAGAGAGGACTGTG | qPCR |
| DUO1R | AGATTTGGGATTGAAACTCG |  |
| DUO3F | CTCTGACGATGAGATGATGGA | qPCR |
| DUO3R | CCAAACTGTGAGATGGTCGTT |  |
| FBL17F | CACCAGGCTACAATCAAAAG | qPCR |
| FBL17R | AACCTGTTTTCGGATAGCTC |  |
| CDKA1F | GCACAGCAATCAGAGAAATC | qPCR |
| CDKA1R | CTATGAGAGTGGCAATACGC |  |
| CYCB1F | CTTGACAGTTCCGACTCATT | qPCR |
| CYCB1R | AATAGCCAGTGTGATGCTTG |  |
| GCS1F | GGATGGTTATGTTTGGTCTT | qPCR |
| GCS1R | GATGGTGATTATGGTGATGA |  |
| UBQ5F | GGTGCTAAGAAGAGGAAGAAT | qPCR |
| UBQ5R | CTCCTTCTTTCGGTAAACGT |  |
| P1 | GAACGTGACTCACAAAGGCGG | genotyping |
| P2 | CTGCCAAGTCTTTTCACAAGC |  |
| P3 | GGAGAGGAGACTGGAGCAGA | RT-PCR |
| P4 | AGCCTTGGTTTGTTCTTTGG |  |
| miR159aF2 | GGCTTTTACTCTTCTTTGGATTGA | qPCR |
| miR159aR2 | CACGCTAAACATTGCTTCGGA |  |
| miR159bF2 | ATGGCTTCACTCTTCTTTGGATTG | qPCR |
| miR159bR2 | CCTACTCAAGATCCATCATCCAT |  |
| miR159cF2 | AGCTCCTTTTCTTCTTCTCTTAAT | qPCR |
| miR159cR2 | CGTCTTCTCGTAAATAAACAACATT |  |
| AtGP1_F | TGGTTTTTCCTGTCCAGTTTG | qPCR/ChIP |
| AtGP1_R | AACAATCCTAACCGGGTTCC |  |
| soloLTR_F | AACTAACGTCATTACATACACATCTTG | qPCR/ChIP |
| soloLTR_R | AATTAGGATCTTGTTTGCCAGCTA |  |
| siR02_F | CAATATGTTCTTCACCATCG | qPCR/ChIP |
| siR02_R | ATTTGCGAAACTAATGGAAG |  |
| AtSN1_F | ACCAACGTGCTGTTGGCCCAGTGGTAAATC | qPCR/ChIP |
| AtSN1_R | AAAATAAGTGGTGGTTGTACAAGC |  |
